# Ranging in an Underwater Medium with Multiple Isogradient Sound Speed Profile Layers

**DOI:** 10.3390/s120302996

**Published:** 2012-03-02

**Authors:** Hamid Ramezani, Geert Leus

**Affiliations:** Faculty of Electrical Engineering, Mathematics and Computer Science, Delft University of Technology, 2826 CD Delft, The Netherlands; E-Mail: g.j.t.leus@tudelft.nl

**Keywords:** localization, ranging, sound speed profile, ray tracing, underwater acoustic sensor networks

## Abstract

In this paper, we analyze the problem of acoustic ranging between sensor nodes in an underwater environment. The underwater medium is assumed to be composed of multiple isogradient sound speed profile (SSP) layers where in each layer the sound speed is linearly related to the depth. Furthermore, each sensor node is able to measure its depth and can exchange this information with other nodes. Under these assumptions, we first show how the problem of underwater localization can be converted to the traditional range-based terrestrial localization problem when the depth information of the nodes is known a priori. Second, we relate the pair-wise time of flight (ToF) measurements between the nodes to their positions. Next, based on this relation, we propose a novel ranging algorithm for an underwater medium. The proposed ranging algorithm considers reflections from the seabed and sea surface. We will show that even without any reflections, the transmitted signal may travel through more than one path between two given nodes. The proposed algorithm analyzes them and selects the fastest one (first arrival path) based on the measured ToF and the nodes’ depth measurements. Finally, in order to evaluate the performance of the proposed algorithm we run several simulations and compare the results with other existing algorithms.

## 1. Introduction

Available wireless sensor network (WSN) localization techniques rely on mutual distances between sensors [1], which are for instance estimated from time of flight (ToF) measurements. In homogeneous medium, like air, where the propagating wave speed is constant, the mutual distances between the nodes are linearly related to the ToFs. In contrast, the propagating wave speed inside an inhomogeneous medium is not constant, and depends on the location. In such a medium, the ToF between two nodes depends not only on the sound speed profile (SSP) of that medium but also on the position of the two nodes [2]. Therefore, the ToF is not linearly proportional to the Euclidean distance between the nodes, and the distance-dependent localization techniques are not appropriate for inhomogeneous media. As a result, they should be modified to ToF-based techniques.

In [3], the problem of localizing a node in an underwater environment with a known depth-dependent SSP is considered. As the target node measures the ToF from an anchor node, the corresponding constant range interval surface for the measured ToF is constructed. To construct a single constant range interval surface, the path trajectory for each departing ray from an anchor node is calculated. Then on each path trajectory, a point is selected based on the measured ToF. All theses points together form the constant range interval surface. After sufficient ToF measurements are taken, the position of the target node is estimated as a point whose sum of squared distances from all these surfaces is minimum. The main drawback of this approach is the computational complexity which is dependent on the network size and the required accuracy.

In our earlier work [4], we consider the problem of localizing a target node in an underwater environment with an isogradient SSP. There, we directly work with the ToF measurements, and we localize a target node based on the ToF equations. Since the algorithm is based on an analytical approach, the computational complexity is acceptable. Although the assumption of a single isogradient SSP is appropriate for a deep underwater medium, it is not valid for the entire environment. The sound speed at a given point in an underwater medium is affected by the salinity, water temperature and pressure of that point [5], and in general this causes the SSP to vary nonlinearly and even non-monotonically with respect to (w.r.t.) the depth, especially in a shallow underwater medium.

In [6] and [7], the localization of an underwater WSN is investigated, in a 3D environment assuming knowledge of the nodes’ depth. These algorithms can help us to convert the inhomogeneous underwater localization problem into a traditional 2D homogeneous distance-dependent localization problem, which is well-studied in the literature [8,9]. We will show that as the depth information is known a priori, the ToF between two nodes only depends on the horizontal distance and thus also on the distance between the nodes. Hence, having the ToF measurement between two nodes, the pair-wise distance can be computed. As an example, based on the depth information and the SSP, a look up table (LUT) is built in [10], which relates the ToF measurement to the horizontal distance between two nodes. The algorithm of [10] is very fast, but to scan the whole inhomogeneous environment, a huge LUT is required which may not be practical. Furthermore, the SSP in an underwater medium is subject to changes in temperature and conductivity, and any change in SSP degrades the LUT accuracy and upsets the localization performance.

In [11], the problem of ranging in an inhomogeneous underwater environment is considered. A numerical range estimator is proposed which is based on reconstructing the slanted path using Fermat’s principle and calculus of variations. Basically, after depth and time measurements, the authors form an integral equality which is taken over the depth between nodes. Then, they try to numerically calculate the constant defined by Snell’s law. Afterwards, by using the computed constant, they calculate the horizontal distance between the nodes through another integral equality. Their work is really comprehensive since with any given SSP, the horizontal distance is computable, but their algorithm to compute the constant value (defined by Snell’s law) has an ambiguity, because in an inhomogeneous medium it is common that a traveling ray from one node to another node passes a given depth more than once. Since the depth of a point on a traveling ray trajectory is not a monotonic function of the depth, this phenomenon casts an ambiguity on the value of any integral taken w.r.t. the depth along the traveling path.

In this work, we analyze the acoustic signal propagation between two sensor nodes in an underwater environment. We use a ray-tracing approach to model the propagation which is a valid approximation for high-frequency signal transmission [10]. We assume that the underwater medium is composed of different layers with an isogradient SSP, which is a practical model for the actual SSP of the entire environment [12,13]. We will show that in such an environment, if the depth information of two nodes located on a specific ray is known, then the positions of the crossing points, where the ray trajectory and the layer boundaries meet each other, can be obtained through a set of polynomial root finding equations. Based on these equations we are able to distinguish among different possible transmission paths between the nodes, and determine the fastest one. The proposed method for finding the fastest transmission path between the nodes can handle reflections from the surface and the seabed by adding more polynomial equations to the set. Another contribution of this paper is a novel method for accurate ranging between the nodes. The proposed algorithm computes the horizontal distance between two nodes based on the ToF and depth measurements. The algorithm estimates the range of a target by minimizing the difference between the measured ToF and the constructed ToF estimated from the known map in an iterative manner.

The rest of the paper is organized as follows. We describe the network model in Section 2, and we compute the ToF *versus* the node positions in Section 3. Next, in Section 4, we propose our ranging algorithm, and we extract its CRB. Then in Section 5, we evaluate the performance of the proposed algorithm through several simulations. Finally, we conclude the paper in Section 6.

## 2. Network Model

Consider *K* anchor nodes with known locations and one target node in an underwater acoustic sensor network (UASN). The goal of the system is to estimate the position of the target node with ToF and depth measurements. To relate the wave ToF to the node position inside an underwater medium, we are faced with the classical problem of how an individual ray behaves in the medium, and how a ray departing one node arrives at the other node.

Assume that the wave speed in a Cartesian coordinate system is a function of the position and is defined by *c*(*x, y, z*). Then, we can compute the ToF between two nodes, **x**^S^ and **x**^E^ as

(1) $$t=\int_{s(x^{S},x^{E})}\frac{\mathrm{ds}}{c(x,y,z)}$$

where *s* is the arc length, which is related to the ray path according to the standard ray equation

(2) $$\frac{d}{\mathrm{ds}}\;\left(\frac{1}{c(x,y,z)}\;\frac{dx}{\mathrm{ds}}\right)=-\frac{1}{c^{2}(x,y,z)}\;\nabla c(x,y,z)$$

with **x** = **x**(*s*) = [*x*(*s*), *y*(*s*), *z*(*s*)]*^T^* the position on the ray determined by the arc length *s*. It is clear from Equation (1) that in an inhomogeneous medium the ToF between two nodes is not linearly dependent on the distance between them, and is a function of the two node positions. Using the ToF measurements to *K* anchors, the location of a node can be obtained as,

(3) $$\hat{x}=\underset{x}{\text{min}}\;{\left|t(x)-t_{m}\right|}^{2}$$

where **t**(**x**) is a *K* × 1 vector with the *k*-th element representing the ToF between the target node and the *k*-th anchor node, and **t**_m_ is a vector denoting the noisy ToF measurements to all *K* anchors. To localize a node in a 3D inhomogeneous environment without depth information at least four anchors are needed, and the localization process is known as quadrilateration. If the depth information is available, it is possible to localize a node with just three anchors, but still we have to work with ToFs. For more information about how the anchor nodes inside the network are selected and how they can communicate with each other, the reader is referred to [6].

In an underwater medium where the sound speed varies only with depth, the availability of depth information does not only allow us to work with only three anchors, it also opens the door to convert the time-based localization problem into a traditional range-based one as explained next.

Since the SSP is only a function of depth, the problem of ray tracing between two nodes has a cylindrical symmetry around the line parallel to the *z*-axis and passing through one of the nodes. Hence, we can map the problem of ray tracing into a vertical plane that crosses the two nodes. As the depth of each node inside the plane is known, the ToF only depends on the horizontal range between the nodes. In other words, the horizontal distance between the nodes is the only variable that determines the ToF. Suppose now that the ToF of the first arrival path as a function of the horizontal distance is an invertible function, meaning that the ToF of the first arrival path between two points located at specific depths is a monotonic (actually increasing) function of the horizontal distance between the nodes. Then, one parameter can be computed from the other, and we are basically able to estimate the distance between the nodes using the corresponding ToFs. If the above assumption does not hold for a given environment, then there would be ambiguities in the ranging problem. However, this monotonicity is generally observed.

**Figure 1.**
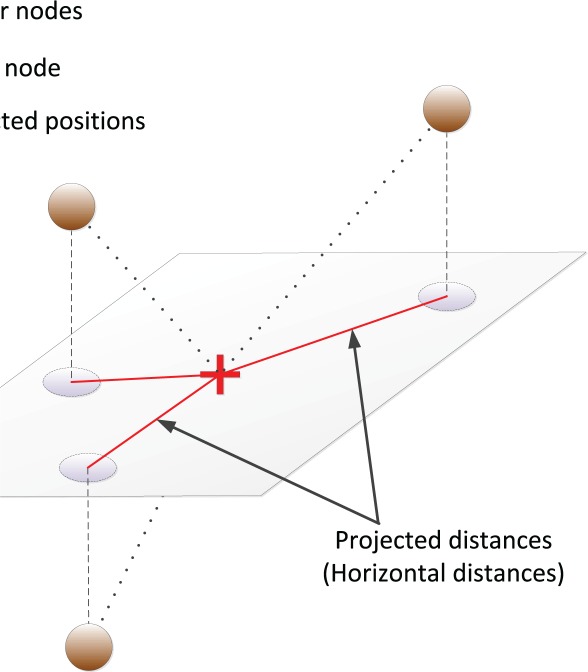
Projection of pair-wise distances on the horizontal plane crossing the target.

The conversion of a 3D underwater localization problem to a 2D one can now be explained as depicted in Figure 1. Using the ToFs and depth information, the unknown node computes the pair-wise distances to the other nodes. Then it projects the estimated distances on a horizontal plane at its depth. Finally, based on the projected distances, 2D multilateration is performed to compute its position [7]. Therefore, localizing a node in this case only requires the knowledge of the projected distances to the anchors or the estimated horizontal distances.

## 3. ToF *Versus* Node Positions

In order to relate the ToF to the node positions, we first require to find which ray departing the source reaches a specific destination. In this section, we analytically find the rays that can travel between two nodes with known positions, and we compute their corresponding ToFs based on their trajectories. It is worth mentioning that in an underwater medium with fixed SSP, each ray departing a source can be uniquely characterized by its departing angle.

Here the SSP is considered as a piece-wise linear function of the depth which is a valid approximation according to the measured data [14]:

(4) $$c^{\left(j\right)}\;(z)=a^{\left(j\right)}\;z+b^{\left(j\right)},\;z^{\left(j-1\right)}<z<z^{\left(j\right)},\;j\in 1,\ldots ,N$$

where *z* represents the depth, *a*^(*j*)^ and *b*^(*j*)^ are related to the chemical and physical characteristics of the *j*-th isogradient SSP layer, and *N* is the number of layers. In our previous work [4], we show how a ray can travel between two nodes located inside an isogradient SSP underwater environment. We review this work first for completeness.

### 3.1. ToF *Versus* Node Positions in a Single Layer

#### Exact Propagation

**Figure 2.**
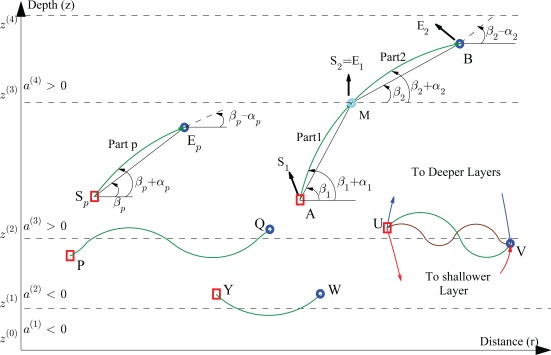
Samples of ray trajectories as they travel through different layers.

In a single layer, each truncated ray (indexed by *p*) between two points, *i.e.*, S*_p_* and E*_p_* in Figure 2, can be uniquely characterized if the position of the starting point, position of the end point, and SSP are known. In order to simplify the notation, we index the SSP from now on by the truncated ray index instead of the layer index. For instance, for the *p*-th truncated ray located at the *j*-th layer, we introduce the new notation

(5) $$c_{p}\;(z)=a_{p}\;z+b_{p}=c^{\left(j\right)}\;(z)=a^{\left(j\right)}\;z+b^{\left(j\right)}$$

The relation between the ToF and the node positions can then be extracted from a set of differential equations characterized by Snell’s law [10],

(6) $$\frac{\text{cos}\;\theta }{c_{p}\;(z)}=\frac{\text{cos}\;\theta_{p}^{S}}{c_{p}\;(z_{p}^{S})}=\frac{\text{cos}\;\theta_{p}^{E}}{c_{p}\;(z_{p}^{E})}=k_{0},\;\;\text{and }\theta \in \;\left(\frac{-\pi }{2},\;\frac{\pi }{2}\right)$$

where $\theta_{p}^{S}$ and $\theta_{p}^{E}$ are the ray angles at the starting and end points, respectively, $z_{p}^{S}$ and $z_{p}^{E}$ represent the depth of the starting and end node, respectively, and *k*_0_ is constant along a ray traveling between the nodes (see Figure 2). Moreover, the parameters *θ* and *z* represent the angle and depth of a given point along the ray. Now, we can write

(7a) $$\partial r=\frac{\partial z}{\text{tan}\;\theta }$$

(7b) $$\partial s=\frac{\partial z}{\text{sin}\;\theta }$$

(7c) $$\partial t=\frac{\partial s}{c_{p}\;(z)}$$

where *s* is the arc length of a ray traveling between the two nodes, and *t* is its corresponding travel time. From Equations (5) and (6), by taking derivatives w.r.t. *z* and *θ*, we can write

(8) $$\partial z=-\frac{1}{a_{p}\;k_{0}}\text{sin}\;\theta \;\partial \theta$$

Using the above differential equations, for the two points we have [4]

(9a) $$r_{p}^{E}-r_{p}^{S}=\sqrt{{\left(x_{p}^{E}-x_{p}^{S}\right)}^{2}+{\left(y_{p}^{E}-y_{p}^{S}\right)}^{2}},$$

(9b) $$X_{p}=\frac{z_{p}^{E}-z_{p}^{S}}{r_{p}^{E}-r_{p}^{S}}$$

(9c) $$Y_{p}=\{\begin{array}{ll} \frac{K_{p}}{X_{p}}, & z_{p}^{S}\neq z_{p}^{E}\\ \frac{0.5a_{p}\;\left(r_{p}^{E}-r_{p}^{S}\right)}{b_{p}+a_{p}\;z_{p}^{S}}, & z_{p}^{S}=z_{p}^{E} \end{array}$$

(9d) $$K_{p}=\frac{0.5a_{p}\;\left(z_{p}^{E}-z_{p}^{S}\right)}{b_{p}+0.5a_{p}\;\left(z_{p}^{E}-z_{p}^{S}\right)},$$

(9e) $$\text{tan}\;\beta_{p}=X_{p}$$

(9f) $$\text{tan}\;\alpha_{p}=Y_{p}$$

(9g) $$\theta_{p}^{S}=\beta_{p}+\alpha_{p}$$

(9h) $$\theta_{p}^{E}=\beta_{p}-\alpha_{p}$$

(9i) $$t_{p}=\frac{-1}{a_{p}}\;\left[\text{ln}\;\frac{1+\text{sin}\;\theta_{p}^{E}}{\text{cos}\;\theta_{p}^{E}}-\text{ln}\;\frac{1+\text{sin}\;\theta_{p}^{S}}{\text{cos}\;\theta_{p}^{S}}\right]$$

where $r_{p}^{E}-r_{p}^{S}$ is the horizontal distance between the points, $x_{p}^{S}={\left[x_{p}^{S},\;y_{p}^{S},\;z_{p}^{S}\right]}^{T}$ and $x_{p}^{E}={\left[x_{p}^{E},\;y_{p}^{E},\;z_{p}^{E}\right]}^{T}$ are the coordinates of the starting and end points, respectively, and *t_p_* is the traveling time of a truncated ray between these two nodes. Note that *β_p_* represents the angle of the straight line connecting the nodes w.r.t. the horizontal axis, and *α_p_* is the angle between the actual ray and this straight line. From Equations (9g) and (9h), it can be seen that the ray deviations from the straight line at the starting and end point are the same but have opposite signs.

#### Linear ToF Approximation

The simple linear ToF approximation based on the depth information between two nodes in the same layer, which assumes a straight-line ray propagation, can be derived as

(10) $$t_{\text{app},p}=\int_{z_{p}^{S}}^{z_{p}^{E}}\frac{1}{\text{sin}(\beta_{p})}\;\frac{\mathrm{dz}}{c_{p}\;(z)}=\frac{d^{S_{p}E_{p}}}{z_{p}^{E}-z_{p}^{S}}\;\frac{1}{a_{p}}\;\text{ln}\;\left(\frac{c_{p}^{E}}{c_{p}^{S}}\right)=\frac{d^{S_{p}E_{p}}}{c_{p}^{E}-c_{p}^{S}}\;\text{ln}\;\left(\frac{c_{p}^{E}}{c_{p}^{S}}\right)$$

where *d*^S_*p*_E_*p*_^ is the straight-line distance between the starting point and end point of the truncated ray, and $c_{p}^{S}$ and $c_{p}^{E}$ are the sound speeds at the starting and end point, respectively. From Equation (10), it can be seen that *t*_app*,p*_ has a linear dependency on the range which is analogous to homogeneous medium. A similar approximation for a multi-layer medium which assumes a straight-line wave propagation between two nodes, **x**^S^ and **x**^E^, can be obtained as

(11) $$t_{\text{app}}=\frac{d^{\text{SE}}}{z^{E}-z^{S}}\sum_{p=1}^{P}\frac{1}{a_{p}}\;\text{ln}\;\left(\frac{c_{p}^{E}}{c_{p}^{S}}\right)$$

where *d*^SE^ is the distance between the nodes, and *P* is the number of single-layer parts of the straight ray. For a straight ray, the number of single layer parts between two nodes is exactly the same as the number of layers it passes. The accuracy of the straight-line approximation will be evaluated in the numerical section.

**Figure 3.**
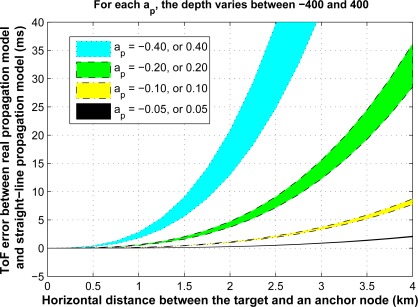
ToF error of the straight-line propagation model in a single layer for different values of range and depth.

Fermat’s principal, which also leads to Snell’s law, states that the path traveled by a ray between two points is the path that can be traversed in the least amount of time. Therefore, the approximated time based on a straight-line ray propagation, *t*_app_, is always greater than the actual ToF. For instance, in Figure 3, it is shown that the difference between *t*_app*,p*_ and the actual ToF, *t_p_*, is always positive. Here, we assume that the sound speed at *z* = 0 is *b_p_* = 1,480 m/s, and we compute the ToF error for different values of depth, distance, and sound speed steepness. It is shown that as the absolute value of the SSP steepness, |*a_p_*|, increases this error grows exponentially. In addition, for the low values of SSP steepness, the change in depth has less effect on this error in comparison with the change in pair-wise distance.

### 3.2. ToF *Versus* Node Positions for Two Adjacent Layers

We start our analysis by considering a ray traveling between two points in adjacent layers. As illustrated in Figure 2, there are many ways for a ray to travel from U to V. An analysis of all possible rays is feasible, and we will later on discuss this. Now, we only focus on a ray which crosses the intermediate boundary only once, and does not propagate into other layers except these two adjacent layers such as the ray from A to B in Figure 2. In this scenario, when the *r* coordinate of the crossing point, M, is computed, we are able to relate the positions of the two nodes to the ToF. It can be seen that the ray has two parts, one indexed by *p* = 1, and the other by *p* + 1 = 2. The ending point of the first part is the starting point of the second part. Thus, the two parts of the ray can be related to each other according to

(12) $$\theta_{2}^{S}=\theta_{1}^{E}$$

Another representation for Equation (12) can be obtained by taking the tangent from both sides of the equation. Using Equations (9e) to (9h), the boundary equation can then be modified to

(13) $$\frac{X_{2}+Y_{2}}{1-K_{2}}=\frac{X_{1}-Y_{1}}{1+K_{1}}$$

For a two-part ray, the combination of Sub-Equations (9b), (9c), and (9d) for each part, together with boundary Equation (13) forms a third-order polynomial root finding problem where the roots represent the possible *r* coordinates of the crossing point M. Notice that the node positions and the depth of M are known and as a result, the parameters *K*_1_ and *K*_2_ can be computed easily, *X*_1_ (*X*_2_) is inversely related to *Y*_1_ (*Y*_2_), and the only unknown parameter is *r*^M^ which determines *X*_1_ and *X*_2_. Since there are at most three roots for a third order polynomial, there are at most three ways for a ray departing at A to reach B (note that we are still assuming that a ray crosses the boundary once, and propagates only in these two adjacent layers). For each of these possible rays the ToF can be calculated as,

(14) $$t=\frac{-1}{a_{1}}\left[\text{ln}\;\frac{1+\text{sin}\;\theta_{1}^{E}}{\text{cos}\;\theta_{1}^{E}}-\text{ln}\;\frac{1+\text{sin}\;\theta_{1}^{S}}{\text{cos}\;\theta_{1}^{S}}\right]+\frac{-1}{a_{2}}\;\left[\text{ln}\;\frac{1+\text{sin}\;\theta_{2}^{E}}{\text{cos}\;\theta_{2}^{E}}-\text{ln}\;\frac{1+\text{sin}\;\theta_{2}^{S}}{\text{cos}\;\theta_{2}^{S}}\right]$$

### 3.3. Pattern Definition for Multi-Layer Ray Propagation

To simplify the multi-layer analysis, we define the concept of *ray pattern*. A ray pattern is a set consisting of all possible rays that can travel between two points. For example, a ray pattern of 2.1.1.2 means that, the ray departs the starting point from the second layer, goes to the first layer, hits the surface, and arrives to the second node in the second layer. Therefore, a *ray pattern* has several properties. First, the number of digits used in the *ray pattern* indicates the number of single-layer parts a ray consists of. Second, it shows in which layer each part of a ray is located. Third, the reflection from the sea surface and the seabed can easily be modeled by this concept. Using the *ray pattern* concept, we are able to show how a ray can travel in a given medium, and which pattern may host the fastest ray.

### 3.4. ToF *Versus* Node Positions According to a Given Pattern

The procedure of ToF computation, as a function of the node positions for a ray which has multiple single-layer parts is the same as the two-part ray, but with more boundary equations. The combination of all these equations may form a higher order polynomial root finding problem, which consequently may increase the number of ways that a ray can travel between the two points. To predict how a ray may travel inside a multi-layer underwater area we introduce several lemmas bellow.

**Lemma 1:** The sound speed in a layer with zero steepness SSP is constant. Thus, the wave propagation inside that layer is along a straight line, and for that reason the parameter *α_p_* for each truncated ray at that layer is zero. In this case, the relation between the ToF and the node positions is modified into

(15) $$t_{p}=\frac{1}{b_{p}}\sqrt{{\left(r_{p}^{E}-r_{p}^{S}\right)}^{2}+{\left(z_{p}^{E}-z_{p}^{S}\right)}^{2}}$$

**Lemma 2:** Rays are bent toward the region where the sound speed is lower.

**Proof:** If we place Equation (9b) into Equation (9c), for two nodes at different depths we obtain

(16) $$Y_{p}=\frac{0.5a_{p}\;\left(r_{p}^{E}-r_{p}^{S}\right)}{b_{p}+0.5a_{p}\;\left(z_{p}^{E}-z_{p}^{S}\right)}$$

The denominator of Equation (16) is the average of the sound speed at the two points and is always positive, and hence using Equation (9f) we understand that the angle *α_p_* has the same sign as *a_p_*. When *a_p_* is larger than zero, the ray angle at the starting point is greater than the ray angle at the end point or any other point along the ray trajectory. Therefore, the ray angle has a tendency to become smaller and to bend up where the sound speed is lower. On the other hand, when *a_p_* is smaller than zero, the ray angle has a tendency to become larger and to bend down where again the sound speed is lower. Indeed, as depicted in Figure 2, within a layer, a ray bends toward the region where the sound speed is lower.

**Lemma 3:** In a layer, the depth along a truncated ray between two points, S*_p_* and E*_p_*, can exceed the region $\left[z_{p}^{S},z_{p}^{E}\right]$ if and only if (iff) the sign of $\theta_{p}^{S}\;\theta_{p}^{E}$ is negative. The excess value can be computed as,

(17) $$\Delta z=\{\begin{array}{ll} \frac{a_{p}\;z_{p}^{S}+b_{p}}{a_{p}\;\text{cos}\;\theta_{p}^{S}}\left(1-\text{cos}\;\theta_{p}^{S}\right), & \text{if}\;\left|\theta_{p}^{S}\right|<\left|\theta_{p}^{E}\right|\\ \frac{a_{p}\;z_{p}^{E}+b_{p}}{a_{p}\;\text{cos}\;\theta_{p}^{E}}\;\left(1-\text{cos}\;\theta_{p}^{E}\right), & \text{if}\;\left|\theta_{p}^{S}\right|>\left|\theta_{p}^{E}\right| \end{array}$$

**Proof:** In a single layer, the depth of a node along a given part as a function of the angle can be derived from Equation (6)

(18) $$z=\frac{1}{a_{p}}\;\left(\frac{\text{cos}\;\theta }{k_{0}}-b_{p}\right)$$

where $k_{0}=\frac{\text{cos}\;\theta_{p}^{S}}{a_{p}\;z_{p}^{S}+b_{p}}=\frac{\text{cos}\;\theta_{p}^{E}}{a_{p}\;z_{p}^{E}+b_{p}}$ is a positive constant for a given ray. It is obvious that *z* follows the behavior of cos*θ*, and its extremum occurs when *θ* is zero. In other words, the depth of a node on a truncated ray exceeds the region $\left[z_{p}^{S},\;z_{p}^{E}\right]$ iff the signs of the angles at the starting and end points differ from each other. As a result, the excess value can be computed as,

(19) $$\Delta z=\{\begin{array}{ll} {\text{max}}_{\theta }\;z(\theta )-\text{max}\;\left\{z_{p}^{S},\;z_{p}^{E}\right\} & \text{if }a_{p}>0\\ {\text{min}}_{\theta }\;z(\theta )-\text{min}\;\left\{z_{p}^{S},\;z_{p}^{E}\right\} & \text{if }a_{p}<0 \end{array}$$

which leads to Equation (17).

In this way, it can be understood that if Δ*z* for a one-part ray in a single layer is so large that a ray part crosses another layer, the assumption of a one-part ray propagation has to be changed into a three-part ray propagation, and the equations have to be reorganized accordingly (see for instance the ray in Figure 2 traveling between points Y and W).

**Lemma 4:** A ray can travel multiple times between two layers if the SSP has a local minimum between them.

**Proof:** A ray which is capable of traveling periodically between two layers has both maximum and minimum points of depth on its traveling path, as illustrated in Figure 2 between the points P and Q. Therefore, based on Lemma 2, it must bend from the first layer to second one, and from the second layer to the first one. Since a ray has a tendency to bend toward the low speed region, periodic traveling between two layers happens if the lower speed region is located between the two layers. In addition, the assumption of an isogradient SSP for each layer forces the total SSP to have a local minimum value at the boundary of these two layers.

**Lemma 5:** Reflections from the seabed and sea surface can be formulated as a boundary equation. In a shallow underwater environment, it is very common that a traveling ray hits the sea surface or the seabed before reaching the end point. Based on the physical properties of the seabed and the sea surface, the reflection can be formulated as a boundary equation. For instance, if we consider a perfect reflection from the seabed or the sea surface, the boundary equations can be obtained as

(20) $$\begin{array}{c} \beta_{p+1}+\alpha_{p+1}=-(\beta_{p}-\alpha_{p})\\ \beta_{p+1}=-\beta_{p} \end{array}$$

**Figure 4.**
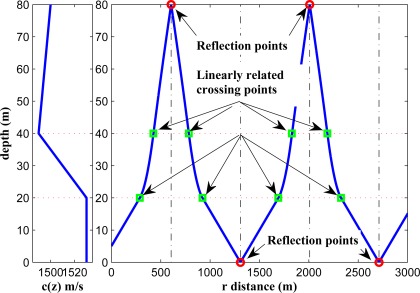
Linear dependency of the reflection and crossing points under the assumption of a perfect reflection.

Under the assumption of a perfect reflection, the reflected parts in different layers have the same properties as the corresponding non-reflected parts but with an axial symmetry around the line parallel to the *z*-axis crossing the reflection point as illustrated in Figure 4. Due to this symmetry which is resulted by the cylindrical symmetry of the ray propagation, the *r*-coordinate of the two crossing points around a reflection point are linearly related to each other, and one can be formulated by the *r*-coordinate of the other. Therefore, this does not change the degree of the polynomial resulting from such kinds of ray patterns.

Thanks to the piece-wise linear behavior of the SSP, we are now able to predict how a ray, which starts from a given point, can travel through different layers to arrive at a specific point. Having built a *ray pattern* using the above lemmas we can then for every *P* part ray relate the ToF to the node’s position using Equation (9) for each single-layer part and the following boundary equations

(21) $$\frac{X_{p+1}+Y_{p+1}}{1-K_{p+1}}=\frac{X_{p}-Y_{p}}{1+K_{p}},\;\;\text{for }p=1\;\text{to }P-1$$

The ToF of any possible *P* part ray can then be computed as

(22) $$t=\sum_{p=1}^{P}\frac{-1}{a_{p}}\left[\text{ln}\;\frac{1+\text{sin}\;\theta_{p}^{E}}{\text{cos}\;\theta_{p}^{E}}-\text{ln}\;\frac{1+\text{sin}\;\theta_{p}^{S}}{\text{cos}\;\theta_{p}^{S}}\right]$$

For instance, as illustrated in Figure 2, four kinds of rays can be predicted between the two points U and V which are described below.

- A ray may travel directly from point U, crossing the boundary once, and not leaving the two adjacent layers. Of course, as formulated before, even with such a limitation, there are at most three paths that can be included in this category.
- A ray may travel directly from point U, crossing the boundary multiple times, not leaving the two adjacent layers. For three crossings of the ray with the boundary, a four-part ray will be obtained. Based on Equation (9c), the *r*-coordinate of the second crossing path is related to the previous one according to
  (23) $$r_{2}^{E}=r_{1}^{E}+2\;\frac{b_{2}+a_{2}\;z_{j-1}}{a_{2}}\;Y_{2}$$
  and similarly,
  (24) $$r_{3}^{E}=r_{2}^{E}+2\;\frac{b_{3}+a_{3}\;z_{j-1}}{a_{3}}\;Y_{3}$$
  and based on the boundary condition we have *Y*_3_ = *−Y*_2_ and *X*_3_ = 0. From the above equations, it can be derived that the crossing points in this scenario are linearly dependent, and therefore the combination of all equations forms a fourth-order polynomial root finding problem owing to the single independent added boundary equation. For the other scenarios with more than three crossing points we have the same analysis, and the problem again reduces to a fourth-order polynomial root finding problem.
- It is also possible that a ray from U to V passes other layers. For instance, in the scenario depicted in Figure 2, the fourth layer has a positive steepness and a ray may bend over at that layer thereby entering a shallower layer. According to the introduced lemmas this is possible, and we should consider it in our analysis.
- Similar to the fourth layer, this phenomenon may also happen in the first layer which forms the fourth category of traveling paths.

**Figure 5.**
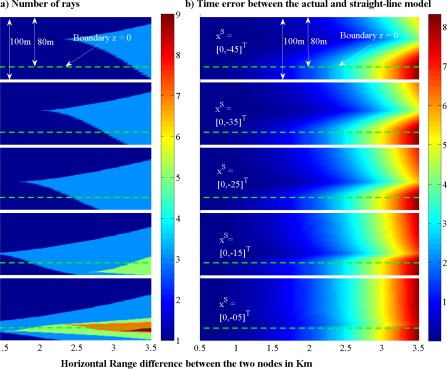
**(a)** Number of paths *versus* the location of the two nodes. **(b)** Range error due to the linear approximation of the ToF.

In Figure 5(a), we show how many rays can travel between two points located in an unbounded two-layer underwater medium. Here, we imagine that the two layers have the same steepness but with different signs, e.g., *a*_1_ = −*a*_2_ = 0.1, and consequently the SSP has a minimum value at the boundary of the two layers. The sound speed at the boundary (*z* = 0) is assumed to be 1,480 m/s. To compute the number of possible rays between two points, we fix the position of **x**^S^, and change the position of **x**^E^ to cover a 100 m by 3.5 km area in the vertical plane as depicted in the figure. According to the discussed lemmas, the possible patterns that can propagate between these two points are 1.2, 1.2.1.2, 1.2.(1.2)…, or 1.1, 1.2.1, 1.(2.1)…, depending on where the two points are located. It is shown that as the pair-wise distance between the two nodes which are located close to the boundary of the two layers increases, the number of paths between them increases too. Around the region where SSP has a minimum value, a pattern with lower number of digits has a lower ToF, but a greater overshoot. Therefore, to compute the fastest ray in this region we can start searching with a simple pattern, and check Lemma 3. If Lemma 3 holds, then we can stop, otherwise we should continue with a more complicated pattern.

For the same scenario as described above, Figure 5(b) shows the error due to a linear approximation of the ToF. From the figure, it can be observed that as the distance between the two nodes gets larger, the linear approximation performs worse. In addition, another effect that influences the accuracy of the straight-line propagation is the angle of this line with the horizontal axis. The larger the angle of the straight line with the horizontal axis, the better the accuracy of the straight-line propagation model.

The above analysis indicates that if a UASN is forced to utilize a straight-line propagation model due to any reason (e.g., system complexity) to achieve more accurate localization results in a noise-free environment, it is suggested that sensors are deployed in such a way that the angles of the straight lines between the nodes become large. However, when noisy measurements exist, we should also consider their influence on the mapped horizontal distances.

## 4. Pair-Wise Underwater Ranging

To our best knowledge, the algorithm in [11] is the only available mathematical approach for underwater range estimation. In order to better understand this algorithm we shortly review it here. The horizontal range between two nodes at different depths in an underwater medium can be obtained from Equations (6) and (7)

(25a) $$t=\int_{z^{S}}^{z^{E}}\frac{1}{c(z)}\;\frac{1}{\sqrt{1-{\left[k_{0}c(z)\right]}^{2}}}\;\mathrm{dz},\;\;\;0<k_{0}<\underset{z}{\text{min}}\;\frac{1}{c(z)}$$

(25b) $$r^{E}-r^{S}=\int_{z^{S}}^{z^{E}}\frac{k_{0}c(z)}{\sqrt{1-{\left[k_{0}c(z)\right]}^{2}}}\;\mathrm{dz}$$

where *k*_0_ is a constant defined by Snell’s law, *t* is the ToF between two nodes, and *r*^E^ − *r*^S^ represents the horizontal distance between them. The estimation of the horizontal distance has two phases; first, by measuring the depth and ToF information, the value of *k*_0_ can be computed numerically from Equation (25a), and second, by substitution of *k*_0_ into Equation (25b), and taking the integral, the value of *r*^E^ − *r*^S^ can be obtained. However, in an inhomogeneous medium, a ray trajectory is not always a monotonic function of the depth, and as a result, whenever a path between two nodes crosses a depth more than once, which is quite common, the above formulas are not valid anymore. In this case, either Equation (25a) has no answer for *k*_0_ in the specified range, or the obtained answer is not valid.

### 4.1. Proposed Algorithm

Assume that, at a specific depth, the ToF of the fastest ray is a monotonic function of the horizontal range. In other words, a propagating wave at a specific depth reaches the destination with a smaller horizontal distance faster. Then, using the ToF and depth measurements, we can find the horizontal distance through a root finding algorithm such as Newton’s method or bisection. Newton’s method is very fast, but it requires the derivative of the ToF w.r.t. the horizontal distance which is hard to compute. The bisection method is robust, and it eventually finds the solution. However, it requires an upper and a lower bound on the horizontal distance. The lower bound can be set to zero, and the upper bound can be computed through multiplying the measured ToF by the maximum sound speed of the entire environment. In spite of the fact that other efficient numerical root-finding algorithms can also be used, we utilize the simple bisection algorithm for the results in the simulation section.

Algorithm 1 shows the steps of the proposed algorithm. In this algorithm, *K* and *E* are the user-defined limits on the stopping criteria that determine when the algorithm exits from the loop, *r*_low_ and *r*_up_ are the lower and the upper bound, respectively. The algorithm starts by initializing the upper and the lower bound on the range, and then it computes the fastest ToF for the midpoint of the bounds. In order to calculate the fastest ToF, given the depth of the two points, different ray-patterns that may host the fastest ray are formed, and all the rays between the points are found and their corresponding ToFs are computed, *i.e.*, in Algorithm 1, *t*^[*l*]^ represents the ToF of the *l*-th found ray between the points. Then, among all these ToFs, the smallest one is selected. Next, based on the computed ToF, the lower, the upper, or both bounds are modified accordingly, and the procedure continues until one of the stopping criteria is met. The important factor that influences the complexity of the proposed algorithm is the number of ray patterns that may host the fastest ray. The ray patterns can be built very efficiently using the proposed Lemmas, still one can add more Lemmas (for a specific SPP) to reduce the number of ray patterns that may host the fastest ray between two nodes.

**Algorithm 1:** Proposed Algorithm.

Compute horizontal distance upper and lower bounds,
*r*_low_ = 0,
*r*_up_ = *t̂c*_max_, where *c*_max_ = max*_z_c*^(*j*)^ (*z*), *j ∈* 1, …, *N*.
Initialize loop parameters,
e = *r*_up_ − *r*_low_,
*k* = 1.
**while** e ≥ *E* and *k* ≤ *K***do**
Compute the average value of the upper and the lower bound, $r=\frac{r_{\text{low}}+r_{\text{up}}}{2}$.
Find the smallest ToF for this horizontal distance - Form all possible ray patterns hosting the fastest ray (see lemmas). - Compute ToF for each possible ray *t*^[*l*]^ (*r, ẑ*^S^, *ẑ*^E^), (see Equation (22)). - Select the ray with the smallest ToF.
*t* = min*_l_t*^[*l*]^ (*r, ẑ*^S^, *ẑ*^E^).
Update the lower or the upper bound,
**if***t < t̂***then**
*r*_low_ = *r.*
**else if***t > t̂***then**
*r*_up_ = *r.*
**else**
*r*_low_ = *r*,
*r*_up_ = *r*.
**end if**
Update loop parameters,
*e* = *r*_up_ − *r*_low_,
*k* = *k* + 1.
**end while**
Compute the estimated horizontal distance between the nodes. ${\hat{r}}^{E}-{\hat{r}}^{S}=\frac{r_{\text{low}}+r_{\text{up}}}{2}$

### 4.2. Cramér–Rao Bound

The Cramér–Rao bound (CRB) expresses a lower bound on the variance of any unbiased estimator of a deterministic parameter. As mentioned before, since the depth information is known and the projection method can be used for localization, a given distance-based traditional localization algorithm works only with horizontal distances. Therefore, in this section we only derive the CRB for the horizontal distance estimation between two nodes. For the computation of the horizontal distance, three measurements are required: two depth measurements which are not directly related to the horizontal distance, and one ToF measurement. It is assumed that all the measurements are affected by Gaussian distributed noise as

(26) $$\begin{array}{lll} \hat{t} & = & t+n_{t}\\ {\hat{z}}^{S} & = & z^{S}+n_{z}^{S}\\ {\hat{z}}^{E} & = & z^{E}+n_{z}^{E} \end{array}$$

where *n_t_*, $n_{z}^{S}$ and $n_{z}^{E}$ are independent Gaussian distributed with variance $\sigma_{t}^{2}$, $\sigma_{z}^{2}$ and $\sigma_{z}^{2}$, respectively. The Fisher information matrix for estimating the horizontal distance (*r*^E^ − *r*^S^), *z*^S^, and *z*^E^ can be obtained as

(27) $$I(r^{E}-r^{S},\;z^{S},\;z^{E})=\frac{1}{\sigma_{t}^{2}}\left[\begin{array}{c} \frac{\partial t}{\partial (r^{E}-r^{S})}\\ \frac{\partial t}{\partial z^{S}}\\ \frac{\partial t}{\partial z^{E}} \end{array}\right]\;\;\left[\begin{array}{ccc} \frac{\partial t}{\partial (r^{E}-r^{S})} & \frac{\partial t}{\partial z^{S}} & \frac{\partial t}{\partial z^{E}} \end{array}\right]+\left[\begin{array}{ccc} 0 & 0 & 0\\ 0 & \sigma_{z}^{-2} & 0\\ 0 & 0 & \sigma_{z}^{-2} \end{array}\right]$$

**Figure 6.**
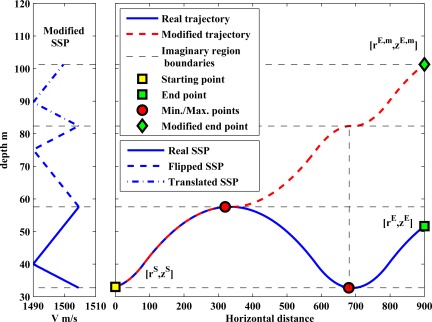
Changing the real ray trajectory into a trajectory which is a monotonic function of the depth.

In order to compute the partial derivative $\frac{\partial t}{\partial (r^{E}-r^{S})}$, we modify the environment in such a way that we can compute the horizontal distance as an integral w.r.t. depth. In order to achieve this, we have to convert the horizontal distance and the ToF to monotonic functions of the depth. Therefore, a ray can not have maximum or minimum points on its trajectory w.r.t. the depth. Let us illustrate the proposed idea with an example. Assume that a ray has a maximum point on its trajectory. The ray angle is zero at this maximum point, and after that it changes sign. But, this sign change does not affect Snell’s law, as it is related to the cosine of the ray angle. As a result, we can assume that the ray travels upward instead of downward as depicted in Figure 6, but in a new environment. In this new environment the SSP of each imaginary region must be changed accordingly. For instance, Figure 6 shows that the real SSP is flipped and translated in the first and second imaginary regions, respectively. In other words, the SSPs of the imaginary regions follow the behavior of the modified ray trajectory.

Note that the above conversion can only be done after we compute the fastest ray, because only then we are able to locate the maximum and/or minimum points on the trajectory and build the new environment. Under this assumption, and using Equations (7b), (7b) and (6) we have

(28a) $$t=\int_{z^{S}}^{z^{E,m}}\frac{1}{c^{m}\;(z)}\;\frac{1}{\sqrt{1-{\left[k_{0}\;c^{m}(z)\right]}^{2}}}\mathrm{dz},\;\;\;\;\;0<k_{0}<\underset{z}{\text{min}}\;\frac{1}{c^{m}(z)}$$

(28b) $$r^{E}-r^{S}=\int_{z^{S}}^{z^{E,m}}\frac{k_{0}c^{m}(z)}{\sqrt{1-{\left[k_{0}c^{m}\;(z)\right]}^{2}}}\mathrm{dz}$$

where m indicates that the variable is related to the modified environment. The above equations are similar to Equations (25a) and (25b), hence we can utilize the same approach used in [11] to compute the CRB, which results into

(29) $$\text{var}\left({\hat{r}}^{E}-{\hat{r}}^{S}\right)\geq \sigma_{t}^{2}\;\frac{1}{k_{0}^{2}}+\sigma_{z}^{2}\;\frac{1-{\left(k_{0}c(z^{E})\right)}^{2}}{{\left(k_{0}c(z^{E})\right)}^{2}}+\sigma_{z}^{2}\;\frac{1-{\left(k_{0}c(z^{S})\right)}^{2}}{{\left(k_{0}c(z^{S})\right)}^{2}}$$

## 5. Numerical Results

**Figure 7.**
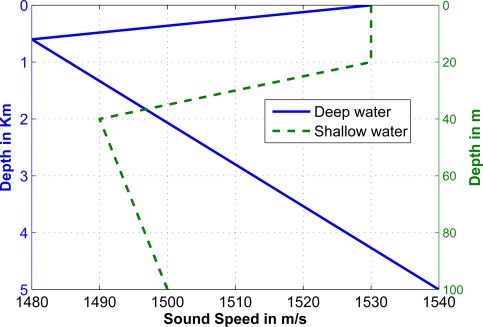
Sound speed profile for deep and shallow water.

In this section we study the performance of finding the fastest path, as well as the proposed ranging algorithm in a multi-layer underwater environment [15]. We consider two kinds of SSPs for our simulations as shown in Figure 7; the former is derived from the sound speed measurements in shallow water [16], and the latter is extracted from the sound speed of the Pacific Ocean and represents a deep water environment [13].

### 5.1. Ray Propagation for Shallow Water

In this part of the report, based on the aforementioned lemmas, we analyze how a ray can propagate between two points inside the shallow water medium. Using the *ray pattern* concept, we are able to show how a ray can travel in a given medium, and which pattern may host the fastest ray. In Table 1, we show the family of patterns a ray may travel between two points through different layers. Since the depth of each node is known, we can select the proper patterns from the table, and form the corresponding polynomial formulas. By finding the roots of the polynomials, the ToF of each ray can be computed, and the fastest one will be recognized.

**Figure 8.**
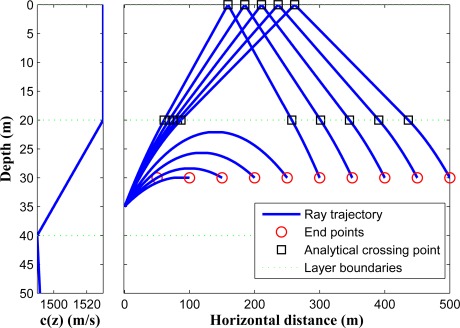
Sample of ray propagation between two nodes.

In Figure 8, we illustrate the ray propagation in a shallow underwater environment between two given points in the second layer. A ray can depart the first point and reach the second point in several ways; (a) it can directly propagate in the second layer without entering the other layers; (b) it can go to the upper layer, hit the surface and go back to the second layer; (c) it can go to the third layer and go back to the second layer; (d) it can go to the third layer, hit the bottom and go back to the second layer. Among all of these possibilities we choose the ray which has the lowest ToF. It is worth mentioning that the algorithm of [11] can not compute the correct horizontal range for any of the drawn blue-colored rays in Figure 8 except for the first one, since all other ray trajectories are not monotonic functions of depth.

**Figure 9.**
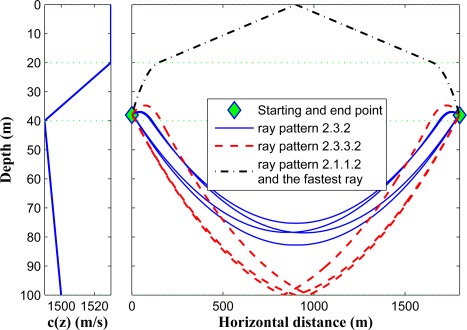
Different possible rays between two points in the second layer.

In Figure 9, we show different possible rays that can travel between two points located in the second layer with a horizontal distance of 1,800 m. Based on the formulation, only three *ray patterns* can exist in this scenario, *i.e.*, 2.3.2, 2.3.3.2, and 2.1.1.2 (here we only consider one reflection from the surface, and only one reflection from the seabed in the existing ray patterns). Since the sound speed has higher values in the first and second layers, the fastest path belongs to the 2.1.1.2 pattern. It can be noted that if the horizontal distance between the two points increases, one *ray pattern* will be eliminated, namely 2.3.2.

### 5.2. Ranging for Deep Water

**Figure 10.**
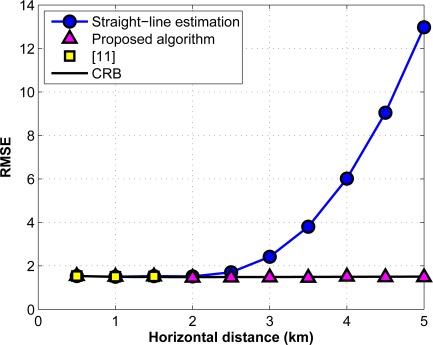
Performance of the proposed algorithm for deep water.

In Figure 10, we compare the performance of the proposed range algorithm with the one introduced in [11], and with the algorithms which approximate the inhomogeneous underwater medium as a homogeneous one with a presumably constant sound speed, *i.e.*, we use a straight-line range computation based on the depth information. In this simulation, we consider Gaussian noise for the ToF and depth measurements with a standard deviation (std) of *σ_t_* = 1 ms and *σ_z_* = 1 m, respectively. In addition, we choose the deep water environment as a communication medium. The communication is between two points from different layers which are located at depth 550 m and 650 m, respectively. The root mean squared error (RMSE) for the horizontal distance estimation is computed by averaging over 1000 Monte Carlo simulations. As illustrated in this figure, the proposed algorithm performs well for all ranges while the algorithm of [11] has no definite solution from a given point as the horizontal range exceeds a given value. Furthermore, the straight-line algorithm degrades as the distance between the points increases.

**Figure 11.**
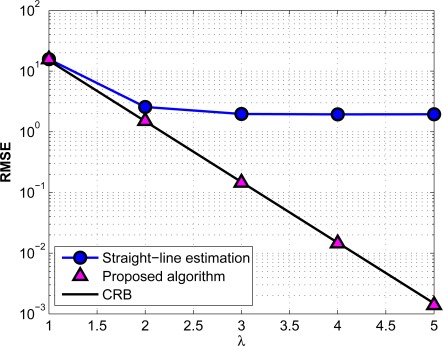
Performance of the proposed algorithm for difference values of noise power.

In Figure 11, we investigate the effect of the measurement noise on the algorithms under consideration. Here the depth of the two nodes is as before, and their horizontal range is fixed at 3 km. The horizontal axis represents how noisy the measured data are. The depth and ToF measurement noise powers are exponentially related to the parameter λ, *i.e.*, *σ_z_* = 10^3^*σ_t_* = 10^2−*λ*^. It can be seen that, the performance of the proposed ranging algorithm constantly improves and attains the CRB when we increase the measurement accuracy, while the straight-line estimation does not show any improvement after a given noise power.

## 6. Conclusions

We have analyzed the problem of localizing a target node in an underwater environment. The inhomogeneous underwater medium upsets the linear dependency of the pair-wise distances to the time of flight. We have shown that, if the depth information of the unlocalized node is available, then the problem of underwater localization can be converted to the traditional range-based one. Dividing the underwater medium into several isogradient sound speed profile layers, we have completely analyzed how a ray can travel between two given points through using different Lemmas. Further, we have proposed an iterative algorithm for the range estimation between two nodes, and we have demonstrated that the proposed algorithm attains the CRB and performs superb in comparison with other existing algorithms. In the future, we want to extend this work for more elaborate SSPs (not necessarily multiple isogradient), especially the ones with one local minimum, for ranging and channel modeling applications.

## Figures and Tables

**Table 1.** All possible patterns that a fastest ray in a shallow underwater environment can follow.

	**to layer 1**	**to layer 2**	**to layer 3**
from layer 1	1	1.2	1.2.3
		1.1.2	1.1.2.3
		1.2.3.2	1.2.3.3
		1.2.3.3.2	1.2.3.2.3

from layer 2	2.1	2	2.3
	2.1.1	2.1.1.2	2.1.1.2.3
		2.3.2	2.3.3
		2.3.2.3.2	2.3.2.3
		⋮	⋮

from layer 3	3.2.1	3.2	3
	3.3.2.1	3.2.1.1.2	3.3
	3.3.2.1.1	3.2.3.2	3.2.3
		3.2.3.3.2	3.2.1.1.2.3
		3.2.3.2.3.2	3.2.3.2.3
		⋮	⋮
